# Effects of alcohol on skeletal muscle contractile performance in male and female mice

**DOI:** 10.1371/journal.pone.0255946

**Published:** 2021-08-12

**Authors:** Joseph A. Laudato, Abigail L. Tice, Jarrod A. Call, Bradley S. Gordon, Jennifer L. Steiner

**Affiliations:** 1 Department of Nutrition and Integrative Physiology, Florida State University, Tallahassee, FL, United States of America; 2 Department of Kinesiology, University of Georgia, Athens, GA, United States of America; University of Tennessee Health Science Center College of Graduate Health Sciences, UNITED STATES

## Abstract

**Background:**

Acute and chronic alcohol use can cause skeletal muscle myopathy in concert with impairments in skeletal muscle strength, function and fatigue resistance. However, the fundamental contractile deficits induced in the presence of alcohol versus those observed in the recovery period following the clearance of alcohol have not yet been characterized nor is it known whether sex influences these outcomes.

**Methods:**

Male and female mice received an intraperitoneal injection of either saline (Control) or ethanol (EtOH; 5g/kg body weight). Muscle force, fatigue, fatigue recovery and twitch characteristics of the posterior crural muscle complex were measured *in situ* 1 hour and 24 hours post alcohol.

**Results:**

In the presence of alcohol (1-hour post treatment) absolute and normalized force generated at 80–150 Hertz was decreased in male and female mice with concurrent reductions in the rate of force development and increases in ½ relaxation time. When expressed as a percentage of maximum force, both males and females also displayed an alcohol-induced leftward shift in the force frequency curve indicative of a type I contractile phenotype. Alcohol enhanced fatigue in both males and females but had no effect on force recovery. Following clearance of alcohol (24-hour post treatment), contractile function was completely restored in females while alcohol treated males experienced sustained reductions in absolute force and had enhanced fatigue compared with male controls.

**Conclusions:**

In the presence of alcohol, both males and females exhibited significant declines in muscle force production and enhanced fatigue; however, following complete clearance of the alcohol, females recovered all functional parameters, while males did not.

## Introduction

The prevalence of alcohol use is rising and in 2019 almost 26% of people older than 18 reported binge drinking in the prior month defined as drinking 5 or more (men) and 4 or more (women) drinks within a few hour time period [1]. Several organ systems and tissues are negatively affected by alcohol consumption, including skeletal muscle. Specifically, alcohol use can impair skeletal muscle contractility leading to losses in muscle strength in a manner that is either independent or dependent on changes in skeletal muscle weight [2–5]. Indeed, classical work in mice and rats has shown several aspects of skeletal muscle contractile physiology, including force production and calcium handling, to be disrupted by alcohol illustrating that the presence of alcohol impacts basic muscle function [6,7].

In contrast, assessment of basic parameters of muscle function (e.g. force, fatigue, etc.) in the recovery period following the complete metabolism and clearance of alcohol are currently limited, despite the potentially detrimental effects a sustained loss in muscle function could have on physical performance in either an athletic setting or for those with physically demanding jobs (e.g. law enforcement, military, construction). In human clinical trials, voluntary muscle force production was reduced in males and not changed in females when assessed in the 24-48hrs after a muscle damaging exercise protocol and subsequent alcohol consumption [2,8,9], suggesting basic muscle contractile parameters may be disrupted by the prior intoxication. In contrast, ex vivo preparations exhibited a complete reversal of alcohol induced impairments in calcium transients when alcohol was removed from the bathing media [2,6,7] and similarly, when the muscle was removed from the alcohol treated animal (acute dose) no decrements in muscle force or fatigue were observed [4]. The contrasting data between voluntary force measures in humans and *ex vivo* muscle preparations could indicate that the maintenance of the physiological environment is an important factor as alcohol may influence things like nerve conduction, blood supply, and the hormonal/metabolic milieu in its manipulation of contractile properties. Therefore, there is a critical gap in our knowledge pertaining to the effects of prior alcohol intoxication on the basic, involuntary contractile capabilities of skeletal muscle within an organism in the recovery period following alcohol intoxication.

Finally, it remains unknown as to whether changes to the basic contractile parameters of skeletal muscle during this recovery period are equally prevalent in males and females. This is important to determine since females exhibit a greater incidence of chronic alcoholic myopathy [10], and therefore may be disproportionately more susceptible to losses in muscle strength, performance and fatigue recovery following acute alcohol treatment. Hence, the purpose of the present investigation was to assess whether the presence of alcohol leads to contractile deficits within the skeletal muscle of male and female mice and to determine whether the expected contractile deficits are sustained during the recovery period after alcohol has been completely metabolized. Presently, we show that the presence of alcohol causes significant decreases in force production and enhances fatigue onset, but that in the recovery period only males continue to exhibit deficits in force and fatigue.

## Materials and methods

### Animals

Male (n = 21) and female (n = 22) C57BL6 mice were purchased from Envigo (Indianapolis, IN) and housed in the Biomedical Research Facility vivarium (Florida State University) for at least 1 week prior to any experimentation. Adult mice used in all experiments were 13–15 weeks of age at the time of the experiment and were randomized to groups of equal body weight within each sex prior to treatment. Mice were housed in a temperature-controlled (25°C) and light-controlled (12:12 light/dark cycle) environment and given *ad libitum* access to water and regular chow diet (LabDiet 5001) at all times during all experiments. All experiments were approved by the Animal Care and Use committee at Florida State University.

### Experimental design

#### Alcohol intoxication

Mice were randomized to either the control (CON) or alcohol (EtOH) group at either the 1 hour or 24 hour time point. As the contractile measurement was non-survival, different animals were tested at 1 hr and 24 hr post injection. EtOH treated mice were given an intraperitoneal (IP) injection of either 5g/kg BW of alcohol (EtOH) or a volume matched injection of saline (CON) and allowed to recover for either 1h or 24h with ab libitum access to food and water. This dose of alcohol is used in mice to study the effects of acute alcohol intoxication and was selected as it had previously been shown to reduce skeletal muscle anabolic signaling (e.g. protein synthesis rates), indicating that the muscle was sensitive to perturbation by alcohol at this dose [3,11]. Despite inherent differences in body weight and fat percentages between male and female mice, peak blood alcohol concentrations and area under the curve calculated over time for alcohol clearance are very similar across sexes when dosing is administered relative to body weight [12]. As further determined by recent work from Pruett et al., 2020 [12], a dose of 5g/kg in mice is translatable to human consumption since doses required to achieve a similar area under the curve of blood alcohol kinetics in mice are ~2-fold higher than in humans.

#### In situ measurement of posterior crural muscle contractility

At either 1h or 24h post-treatment, the posterior crural muscle group (soleus, plantaris, and gastrocnemius muscles) was tested for contractile capacities (Control, n = 5–6 per time point; EtOH, n = 6 per time point). Animals were deeply anesthetized using isoflurane, placed on a 37˚C heated platform, and the distal tendon was attached to a 6650LR servomotor (Aurora Scientific, Aurora, Ontario, Canada) via 4–0 braided silk suture (Roboz Surgical, cat. #SUT-15-2), with the knee secured to a metal screw. Contraction of the posterior crural muscles was elicited via percutaneous stimulation by platinum-iridium electrode needles (#111-725-24TP, Jari electrode supply, Gilroy, CA) on either side of the sciatic nerve using a 701C Bi-Phase Stimulator (Aurora Scientific). Optimal resting length (*L*_*o*_) was determined through a series of twitches each separated by 30 seconds. *L*_*o*_ was defined as the length where the muscle produced the highest active twitch force. Peak-isometric force was defined as the greatest force measured during a 350-ms stimulation using 0.2-ms square-wave pulses at the following stimulation frequencies spaced two minutes apart: 5, 10, 20, 40, 60, 80, 100, 115, 125, and 150 Hz. Fatigability was assessed by 60 isometric contractions produced over a 4-min duration using 350-ms stimulations at 100 Hz (one 350-ms stimulation every 4 sec). After a 5- and 10-minute rest, recovery from fatigue was tested by re-measuring peak-isometric force of the posterior crural muscles. Isometric force-time tracings from the peak pre-fatigue contractions were analyzed to determine rates of contraction and relaxation for the posterior crural muscle group. On two occasions during the stimulation protocol (1 alcohol, 1 control male at 24hr timepoint), the suture or security of the set-up loosened causing erroneous data to be generated. In these instances, the protocol was stopped, and the animal’s data was only included up until this point of “failure”. At the cessation of the testing, the posterior crural muscles from the stimulated leg were excised, weighed, and used to determine normalized force production. Blood was then collected from the inferior vena cava into EDTA coated syringes and the animal was euthanized via cardioectomy while still deeply anesthized. Blood was only collected from animals at the 1 hour time point as unpublished data in our laboratory as well as multiple published reports have shown this dose of alcohol (or higher doses) is completely cleared from the blood by 8-12h post intoxication [12–14]. Blood was centrifuged for 10min at 10,000g for isolation of plasma which was stored at -80˚C until analysis of blood alcohol concentration.

Data were analyzed using the Aurora Scientific ASI 611A Dynamic Muscle Analysis v5.300 software prior to extraction into excel worksheets. The maximum rate of force development was determined as the maximum positive derivative (slope) of the entire force curve beginning from the start of the cursor to the end of the cursor (*cursors* meaning the entirety of the contraction, which included time both shortly before and after stimulation). The half relaxation time began at the pinnacle of peak force and ended when half of that force was measured during relaxation. Both the rate of force development and half relaxation time were calculated from the 125 Hz force trace as both groups reached tetanus at this frequency.

#### Blood alcohol concentration

An aliquot of plasma was thawed for BAC measurement which was performed using the Analox AM1 Alcohol Analyser (Analox Instruments, Toronto, Canada). Plasma samples were run in triplicate and results were averaged.

### Statistical analysis

All data were analyzed using GraphPad Prism Software (San Diego, CA), with significance set at *P*≤0.05, unless stated otherwise. All results shown in bar graphs are presented as mean ± standard deviation (SD), while remaining data are shown as mean ± standard error of the mean (SEM). Blood Alcohol levels at 1-hour post intoxication were compared across sexes using an unpaired, two-tailed Student’s t-test. Within each sex, unpaired, t-tests were used to compare the differences between control or alcohol treated mice for the following measurements: rate of force development, ½ relaxation time and force recovery. Within each sex, force frequency data, as well as fatigue data were analyzed by two-way ANOVA with treatment (CON/EtOH), frequency (Hz) or contraction number as the variables. If significant interactions were detected, Fisher’s LSD test was used for post hoc analysis. P values can be found within the text or the figure legend for all data.

## Results

### Alcohol reduces peak-isometric tetanic muscle force production and induces a type I contractile phenotype in both female and male mice

**Fig 1:**
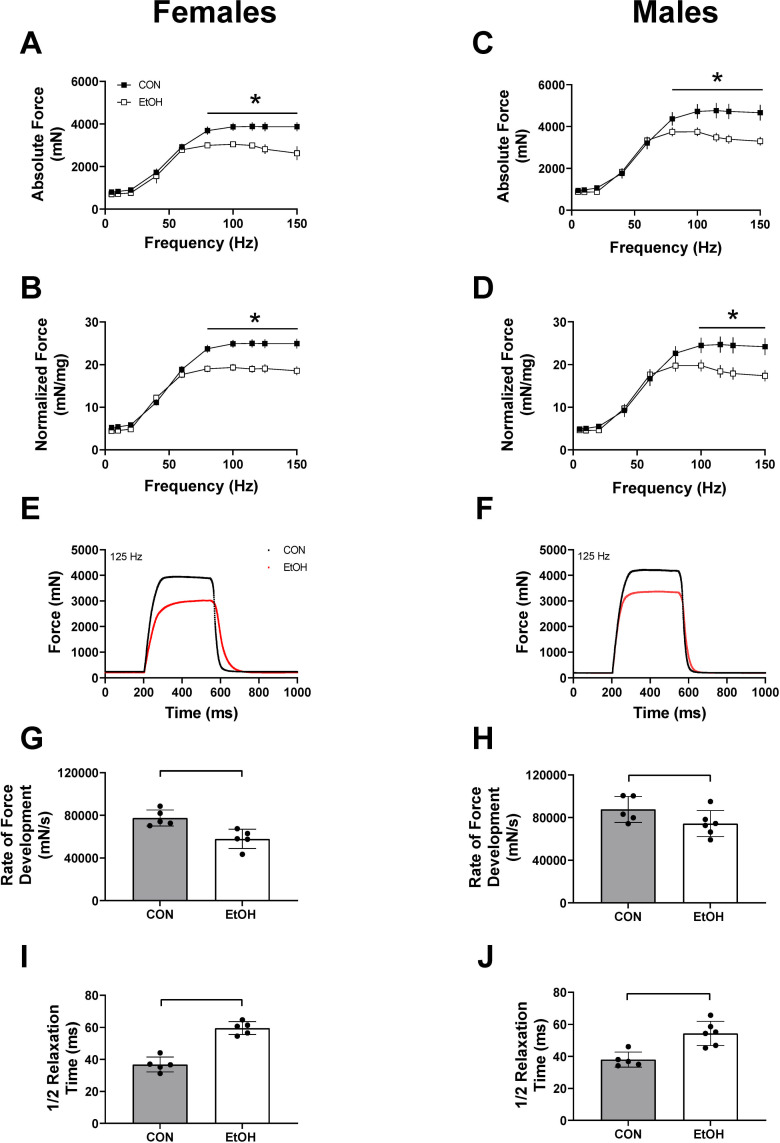
Effects of 1 hour of alcohol intoxication on skeletal muscle force in male and female mice. Absolute force (mN) and force normalized to triceps surae muscle complex weight (mN/mg) during the force frequency protocol at 1 hour for Control (CON) females (n = 5) and Alcohol (EtOH) females (n = 5) (A and B), as well as Control (CON) males (n = 5) and Alcohol (EtOH) males (n = 6) (C and D). Two-way repeated measures ANOVA was used to detected differences between control and alcohol within each sex. Panel A, p = 0.0041; Panel B, p<0.0001; Panel C, P<0.0001, Panel D, p<0.0001. Representative force trace at 125 Hz is shown for females (E) and males (F) at 1 hr. The rate of force development and ½ relaxation time at 125 Hz is shown for females (G, I) and males (H, J). Unpaired T-tests were performed with significance achieved in panel 1G (p = 0.0058), 1H (p = .01045), 1I (p<0.0001) & 1J (p = 0.0024). Data are presented as mean ± SEM for Figures A-D, and as mean ± SD for Figures H-J. * or a line connecting the bars indicates EtOH significantly different than sex-matched CON, (*p*<0.05).

**Fig 2:**
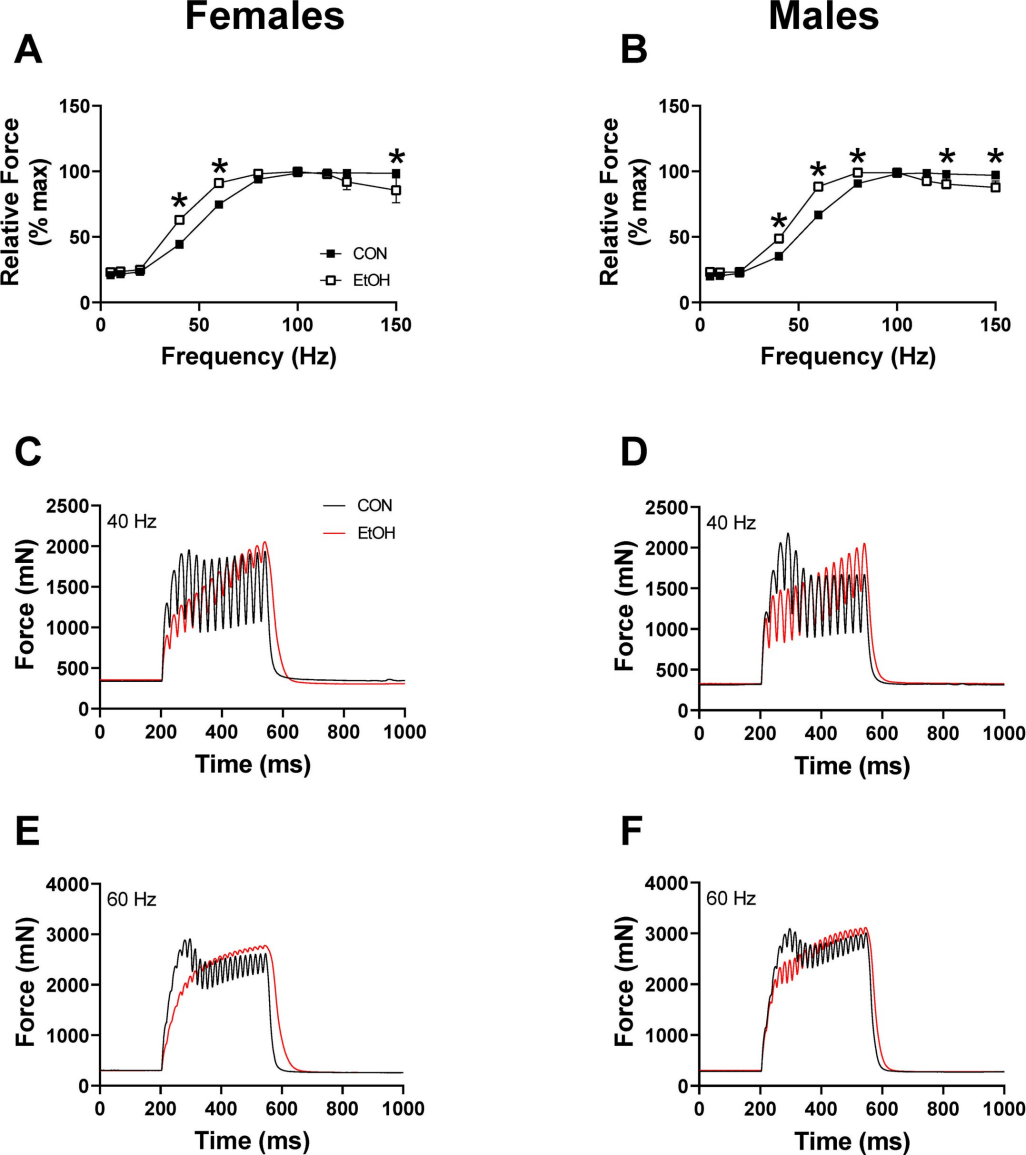
Percentage of maximal force generation 1 hour after alcohol intoxication. The force generated as a percentage of maximal force for females (A) (p≤0.0016), and males (B) (p≤0.0304), 1 hr after alcohol intoxication. Force traces at 40 Hz and 60 Hz for females (C, E) and males (D, F), at 1 hr post alcohol binge. Data in panel A and B were analyzed by 2-way ANOVA and are presented as mean ± SEM. *EtOH significantly different than sex-matched CON at that frequency (*p*<0.05).

One-hour post binge, blood alcohol levels were elevated to a similar magnitude in females (512.4 ± 32.3 mg/dl) and males (555.9 ± 33.6 mg/dl) (p = 0.38). Skeletal muscle weights (soleus, plantaris, gastrocnemius) normalized to body weight within each sex were not different between CON and EtOH treated animals within that sex (S1 Table). Peak-isometric twitch force and associated twitch contractile properties were unaffected by alcohol in either sex at 1 hour (Table 1); however, both absolute and normalized peak-isometric tetanic force were significantly lower following alcohol in both females and males (Fig 1A–1F). Contractile properties determined from peak-isometric force-time tracings (Fig 1E and 1F) suggested an alcohol-induced effect on excitation-contraction coupling as alcohol treatment caused a decrease in the rate of force development in females (Fig 1G and 1H) and a slower ½ relaxation time (Fig 1I and 1J). Analysis of relative force-frequency curves further indicated an alcohol-induced effect on excitation-contraction coupling as there was a leftward shift in both females and males (Fig 2A and 2B). Finally, the summation of twitches was notably greater at 40 and 60 Hz with alcohol treatment (Fig 2C–2F).

**Table 1:** Twitch characteristics for female and male mice 1 hour and 24 hours after acute alcohol intoxication.

	Female - 1hr			Male - 1hr			Female - 24hr			Male - 24hr		
Twitch characteristics	CON	EtOH	*p*	CON	EtOH	*p*	CON	EtOH	*p*	CON	EtOH	*p*
Peak- Isometric force (mN)	818.0 ± 23.2	734.6 ± 42.5	0.123	916.8 ± 41.6	893.9 ± 36.8	0.689	698.2 ± 32.3	741.6 ± 53.2	0.502	919.6 ± 26.3	836.2 ± 20.5	0.036
Normalized force (mN/mg)	5.3 ± 0.2	4.6 ± 0.2	0.065	4.8 ± 0.3	4.7 ± 0.3	0.931	4.71 ± 0.2	5.3 ± 0.4	0.193	5.1 ± 0.3	5.2 ± 0.1	0.398
Time to maximum (ms)	20.1 ± 0.6	20.3 ± 0.5	0.794	19.5 ± 0.6	19.8 ± 0.3	0.703	19.0 ± 0.4	20.08 ± 0.5	0.010	18.9 ± 0.2	19.9 ± 0.4	0.061
Half relaxation (ms)	12.9 ± 0.2	13.3 ± 0.2	0.235	12.9 ± 0.1	12.8 ± 0.1	0.870	12.7 ± 0.3	12.8 ± 0.1	0.666	12.6 ± 0.2	12.7 ± 0.2	0.580

Table 1 lists the values for peak isometric force, normalized force, time to maximum, and half-relaxation time in male and female mice either 1 hour or 24 hours after the initial alcohol intoxication. Data are presented as mean ± SEM.

### Alcohol enhances muscle fatigue, but has little effect on force recovery

**Fig 3:**
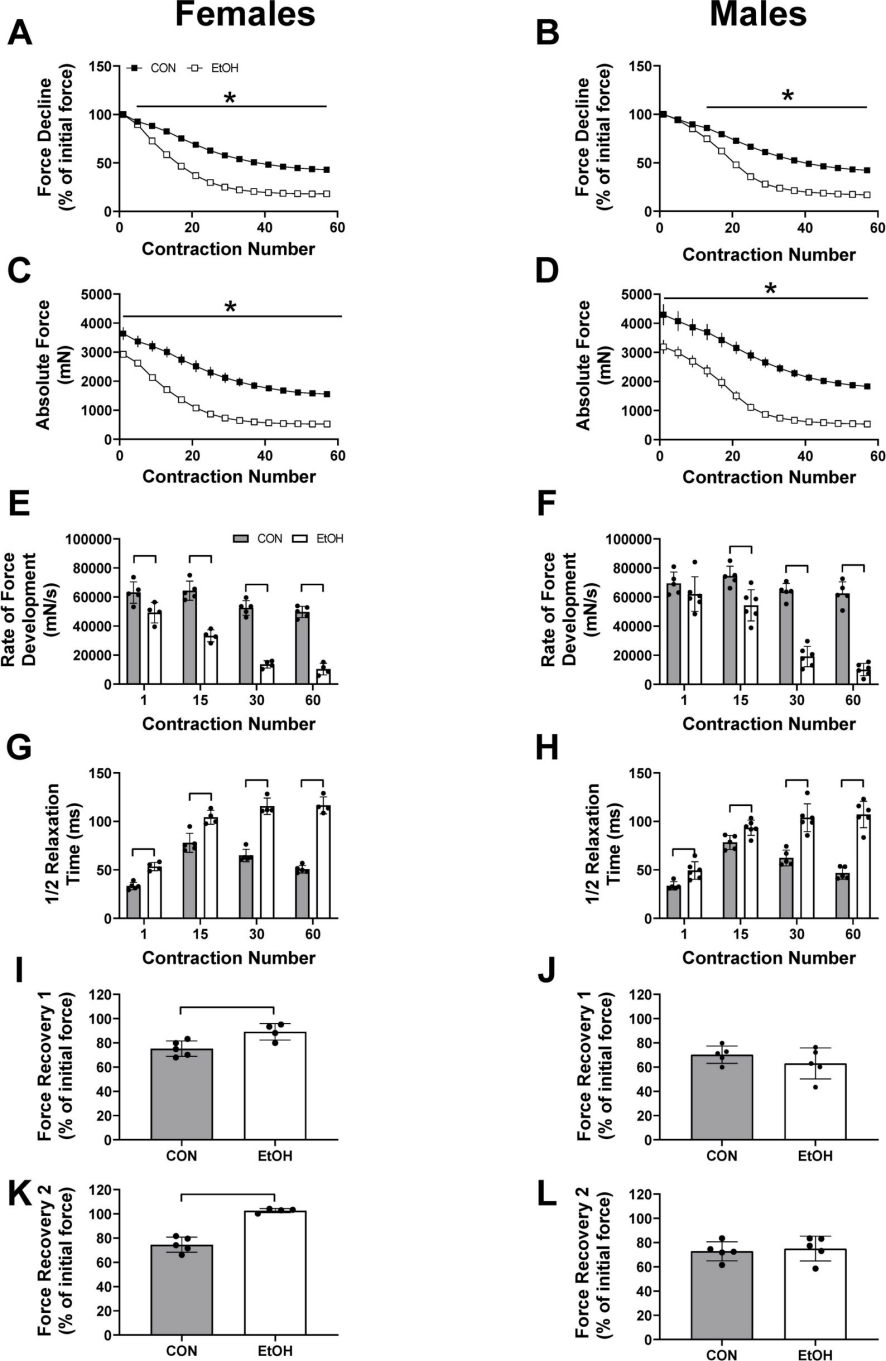
Effects of alcohol intoxication for 1 hour on skeletal muscle fatigue development in male and female mice. Force decline from initial stimulation (%) and absolute force production (mN) during the fatigue protocol at 1 hour for Control (CON) females (n = 5) and Alcohol (EtOH) females (n = 5) (A and C), as well as Control (CON) males (n = 5) and Alcohol (EtOH) males (n = 6) (B and D). The rate of force development and ½ relaxation times at various stimulations (1, 15, 30, 60) during the fatigue protocol are also shown for females (E, G) and males (F, H). Force recovery following a 5-minute rest period shown as a percentage of initial force are shown for females (I, K) and males (J, L). Data were analyzed using two-way ANOVA or unpaired t-tests and are presented as mean ± SE for Figures A-D, and as mean ± SD for Figures E-L. In panels A, B, C, D, p≤ 0.0001 for the main effect of treatment or force, with all also having a significant interaction (A and B, p<0.0001; C, p<0.02), except for panel D. In panels E-K, p<0.05. * or a line connecting the bars indicates EtOH significantly different than sex-matched CON, (*p*<0.05).

Muscle fatigability, as measured by percent loss of force over the course of repeated contractions, was exacerbated by alcohol in both female and male mice (Fig 3A–3D). Contractile properties determined from repeated contraction further distinguished the influence of alcohol on excitation-contraction coupling as alcohol caused a progressive decline in the rate of force development (Fig 3E and 3F) and a progressive slowing in the ½ relaxation time, in both females and males at contraction numbers 1, 15, 30 and 60 (Fig 3G and 3H). Recovery from fatigue was greater at 5 and 10 minutes in alcohol-treated females compared to control females (Fig 3I and 3K), while there was no difference between the male control and male alcohol groups (Fig 3J (p = 0.30) & 3L (p = 0.70)).

### Females recover from alcohol induced contractile dysfunction, while males exhibit lasting effects at 24 hours

**Fig 4:**
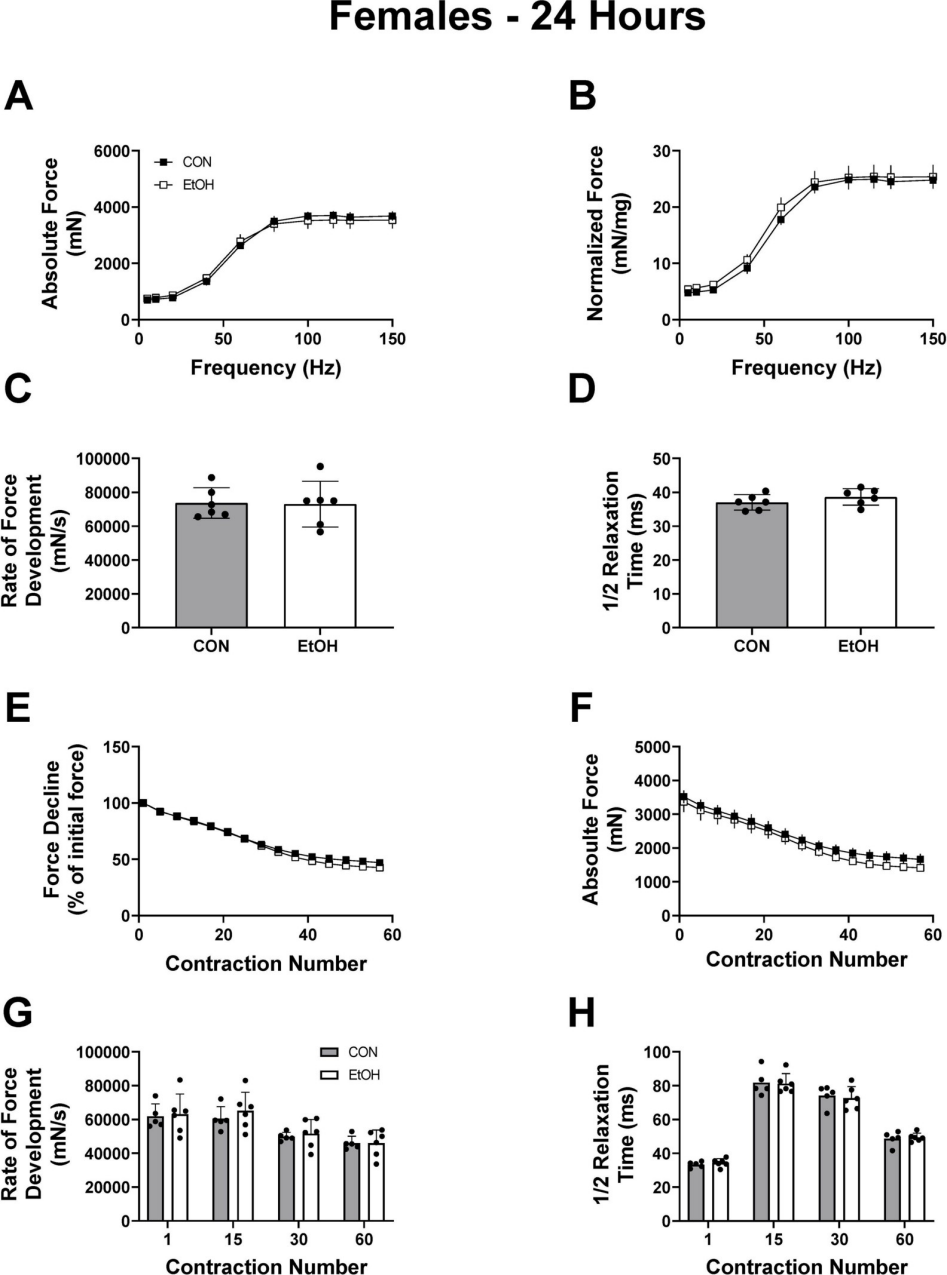
Recovery of muscle contractile properties and fatigue resistance in female mice 24 hours post alcohol intoxication. Outcomes of each contractile test at 24 hours post alcohol binge in female CON (n = 6) and EtOH (n = 6), mice. Panel A and B show absolute and normalized force over a range of frequencies, and Panel C and D show the rate of force development and ½ relaxation time at 125Hz. Panel E and F show the force decline either relative to the first stimulation (E) or in absolute terms (F). The rate of force development at a given contraction number during the fatigue protocol is shown in (G), with the corresponding ½ relaxation time shown in (H). Data were analyzed using two-way ANOVA or unpaired t-tests and are presented as mean ± SEM for Figures A-B and E-F, and as mean ± SD for Figures C-D and G-H.

Contractile measures were repeated 24 hours after the acute binge when alcohol was undetectable in the blood, to differentiate between the effects caused by the presence of alcohol itself, and those incurred by a potentially lasting change in muscle physiology. Skeletal muscle weights normalized to body weight were not different between CON and EtOH treated animals for the soleus, plantaris and gastrocnemius within each sex (S2 Table). Similar to the findings at 1h, there were no differences between control and alcohol for peak-isometric twitch force, normalized force and half relaxation time while time to peak was significantly slower in alcohol treated mice (Table 1). The alcohol-induced decrease in peak-isometric tetanic force over various frequencies that was previously observed at 1h in female mice was no longer apparent at 24h (Fig 4A (p = 0.78) & 4B (p = 0.13)). No differences between groups for contractile properties determined from peak-isometric force-time tracings were detected either (Fig 4C (p = 0.92) & 4D (p = 0.27)). Muscle fatigability and contractile properties during the fatigue protocol at 24h was not different in females between treatment groups as no interactions were observed (Fig 4E (p = 0.99), 4F (p = 0.99), 4G (p = 0.46), 4H (p = 0.99)), however a main effect of EtOH was observed for the decline in force measured during the fatiguing protocol, although the difference in force was negligible between the groups (Fig 4E and 4F, p<0.0001).

**Fig 5:**
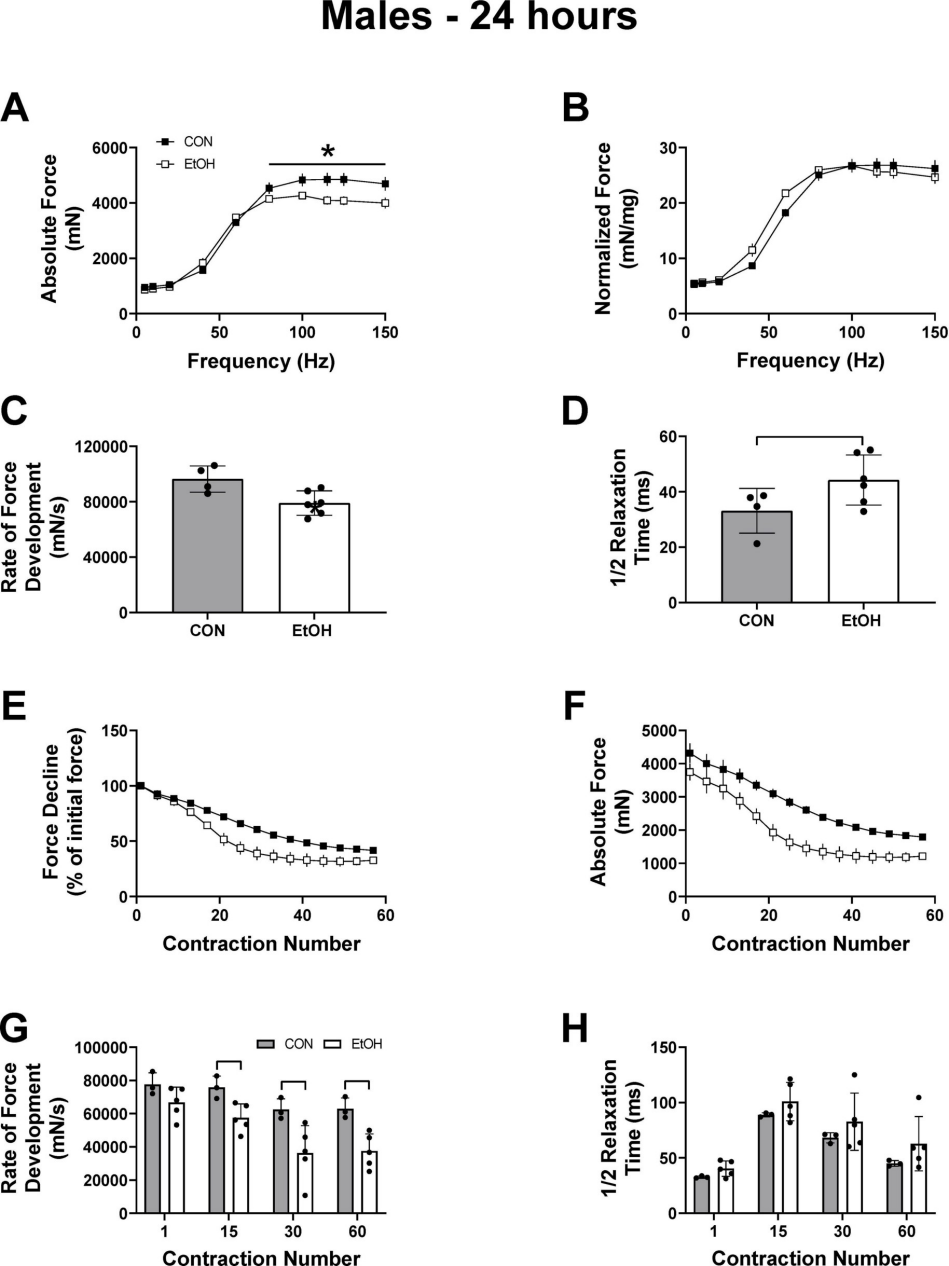
Partial recovery of muscle contractile properties and fatigue resistance in male mice 24 hours post alcohol intoxication. Outcomes of each contractile test at 24 hours post alcohol binge in male CON (n = 3–4) and EtOH (n = 5–6), mice. Panel A and B show absolute and normalized force over a range of frequencies, and Panel C and D show the rate of force development and ½ relaxation time at 125Hz. Panel E and F show the force decline either relative to the first stimulation (E) or in absolute terms (F). The rate of force development at a given contraction number during the fatigue protocol is shown in (G), with the corresponding ½ relaxation time shown in (H). Data were analyzed using two-way ANOVA or unpaired t-test and are presented as mean ± SEM for Figures A-B and E-F, and as mean ± SD for Figures C-D and G-H. * or a line connecting the bars indicates EtOH significantly different than sex-matched CON, (*p*<0.05).

In males, twitch force and contractile characteristics were mostly unaffected 24h post alcohol treatment other than a decrease in peak-isometric twitch force, which was negated when normalized to muscle mass (Table 1). Consistent with this, peak-isometric tetanic force, but not normalized tetanic force, remained lower in the males 24h after alcohol intoxication (Fig 5A (p<0.0001) & 5B (p = 0.31)). Though not as pronounced as at the 1 hour timepoint, males still had a slower rate of force development and a trend for a prolonged ½ relaxation time, indicating a sustained alcohol-induced effect on excitation-contraction coupling (Fig 5C (p = 0.02) & 5D (p = 0.04)). Muscle fatigability remained greater in males subjected to binge alcohol as there was a main effect of treatment detected for both the percentage of force decline and absolute force during the fatiguing protocol (Fig 5E (p<0.0001) & 5F (p<0.0001)). However, no interaction was detected for either outcome (Fig 5E (p = 0.25) & 5F (p = 0.99). The progressive slowing in the rate of force development throughout the fatigue protocol at 24 hours continued to be reduced at stimulation numbers 15 (p = 0.02), 30 (p = 0.04) and 60 (p = 0.009) (Fig 5G, p<0.05). The ½ relaxation time during the fatigue protocol was no longer affected by alcohol at the 24-hour time point (Fig 5H). Recovery from fatigue was also similar between control and alcohol treated mice, in both males and females (S1 Fig). In all, these data show that females were more resilient to a dose of alcohol as males still experienced contractile dysfunction up to 24 hours post alcohol.

## Discussion

Presently, we determined that in the presence of alcohol skeletal muscle fatigue and force production in both male and female mice was significantly impaired. In the recovery period when no alcohol remained in circulation, females had a complete restoration of force and function, while males continued to experience significant contractile dysfunction compared with the corresponding male control group. These data enhance the classical ex vivo work showing alcohol induced impairments in several aspects of contractility, as well as extend them to now include the potentially negative effects of alcohol consumption on muscle contractile performance even in the recovery period, in addition to providing further evidence of a sexually dimorphic response.

The alcohol induced contractile weakness and slow-twitch contractile phenotype are suggestive of an alcohol related impairment in excitation-contraction (EC) coupling. While we cannot completely rule out the possibility that nerve physiology (action potential conduction velocity, acetylcholine release or receptor sensitivity) may have also contributed to the alcohol-induced muscle weakness, the slow-twitch contractile phenotype (i.e. greater twitch summation at lower stimulation frequencies) is not indicative of nerve conduction pathologies. Furthermore, prior work that showed alcohol induced decreases in calcium transients, which underlie EC coupling, was performed using individual muscle fibers that also lacked neuronal innervation, neurotransmitter release or impulse propagation [6,15]. It is also highly unlikely that the slow-twitch contractile phenotypes manifested from an actual fiber-type switch considering the 1-hour timeframe in which alcohol caused these changes. Slow-twitch muscle fiber contractile properties are characterized by slower rates of force generation and relaxation as well as greater twitch summation at sub-maximal stimulation frequencies compared to fast-twitch muscle fibers [16,17]. In healthy muscle fibers, these contractile properties result from the differential expression of slower acting calcium-handling and ATP-hydrolyzing protein isoforms. Presently, there was likely insufficient time for alcohol-induced contractile dysfunction to be a result of fiber type shifting as there was insufficient time for substantial changes to the proteome, and it is therefore more likely that alcohol negatively influenced calcium-handling and ATP hydrolysis properties of the muscle fibers. It is also plausible that these effects were more pronounced in high-force producing fast-twitch fibers considering the exaggerated contractile weakness that was largely reversed at 24 hours.

There are several well characterized changes in calcium flux induced by alcohol that could have contributed to a type II myofiber contractile dysfunction [6,7,15]. For example, alcohol can decrease calcium availability, either via impaired release or decreases in the SR calcium pool, in addition to inhibiting sarcolemmal calcium channels and Ca^2+^ uptake, all of which can culminate in lower contractile strength and slowed relaxation as is presently observed [6,7,15,18,19]. An alcohol induced decrease in calcium availability may also preferentially impede activation of type II fibers as the troponin isoform expressed in type II muscle is less sensitive to calcium compared to the type I isoform, thus requiring more calcium to initiate contraction of type II fibers [20]. Therefore, the presence of alcohol may have changed the contractile ability of the muscle by decreasing calcium flux, however, this is speculative and will require further validation.

Interestingly, at 24 hours post alcohol, muscle fatigue was still enhanced in males, while females had completely recovered, indicating a sexually dimorphic response. This occurred despite reports of an increased propensity for females to develop chronic alcoholic injuries at lower cumulative doses of alcohol [10,21,22]. BAC’s were not different between male and female mice presently, indicating an equal dose of alcohol was administered to each sex. Further, prior work has shown no effect of sex on BAC in mice when dose is administered relative to body weight, indicating that it is unlikely that the rate of alcohol clearance negatively impacted the rate of recovery in males [12,23]. To our knowledge no single clinical trial in humans has directly compared male and female force recovery following alcohol intake alone, although literature does indirectly indicate that females may be more resistant to the effects of alcohol when consumed following muscle damaging exercise (eccentric contractions). In work by McLeay et al. (2017) and by Levitt et al. (2017) [8,9], females did not show any further reduction in the voluntary production of concentric, eccentric and isometric torque 24 hours after alcohol had been consumed following the muscle damaging exercise. In contrast, males experienced a greater loss in peak torque following muscle damaging exercise when alcohol was consumed after the exercise bout (Barnes et al 2010) [2] providing related evidence of another instance of a sexually divergent response to alcohol in the skeletal muscle. Coinciding with these data are other findings at the transcriptional level showing that females have a greater ability to restore cellular homeostasis compared with men following resistance exercise [24]. While resistance exercise and alcohol intoxication likely induce very different transcriptional responses, they can both injure the skeletal muscle and therefore, transcriptional differences could also be contributing to the differing recovery profiles seen presently. Finally, the fiber type composition and therefore contraction speed, energy utilization and substrate metabolism, of male and female skeletal muscle may differ [25,26]. Currently, evidence supports a slightly greater proportion of Type I muscle fibers in female skeletal muscle that coincides with higher oxidative capacity and greater fatigue resistance [25,26]. Alcohol preferentially affects type II myofibers in both chronic settings where muscle atrophy is observed, and acutely when reductions in rates of protein synthesis are quantified [27], indicating that having a slightly larger proportion of Type I fibers may have assisted in the expedited recovery of the female versus male mice following alcohol intoxication.

Lastly, the most apparent possibility explaining the differences in recovery between males and females is differences in concentrations of the various sex hormones and their potential impact on skeletal muscle function. Estrogenic hormones and progesterone have both been shown to enhance muscle contractile properties, and in the case of progesterone, recovery from prior muscle injury in females [28]. Furthermore, loss of estrogen (via ovariectomy), significantly impairs mitochondrial respiratory capacity within skeletal muscle. Testosterone may improve contractile strength via the traditional long-term transcriptional mechanisms given appropriate training stimuli, but also through rapid non-genomic actions that utilize second messenger systems including rapidly modulating intracellular calcium in myotubes [29]. In human subjects, acute alcohol intoxication increased circulating estradiol in women and decreased testosterone in men for at least 24 hrs [30,31]. Moreover, chronic alcohol intake leads to skeletal muscle respiratory dysfunction associated with inhibition of electron transport chain complexes, and an inability to meet oxidative demands during times of heightened ATP usage [32]. But whether acute alcohol intoxication and estrogen interact to influence mitochondrial function and ATP content, and subsequently muscle contractile strength and fatigue, remains an area of future investigation.

Overall, our data indicate that a high dose of alcohol causes significant decrements in intramuscular performance when alcohol is present, and that the majority of those effects are corrected once the alcohol has been metabolized. However, our data also indicate males may be more susceptible to the effects of prior alcohol and may take a longer time to recover force and fatigue resistance, although additional recovery time points will be required to determine how permanent these effects are. Several questions remain that will need to be addressed in future work to determine exactly how the presence of alcohol leads to fatigue and reduces force production, and whether there is a significant impact of the neurological effects of alcohol in relation to skeletal muscle contractility. Finally, the impact of sex hormones as well as the overall dose of alcohol will also need to be explored further to elucidate whether there is a threshold dose for each sex under which no negative effects are observed either acutely or in the post alcohol recovery period.

## Supporting information

S1 Fig Fatigue recovery was not impaired 24 hours after alcohol intoxication. Force recovery 24 hours post-treatment in female (A, B) and male (C, D) mice at 5 minutes (Recovery-1) and 10 minutes (Recovery-2) following the fatigue protocol, presented as a percentage of the initial stimulation. Group sizes are as follows: Females: CON n = 5, EtOH n = 6; Males: CON n = 3, EtOH n = 5. Data are presented as mean ± SD.
(EPS)

S1 Table Skeletal muscle weights made relative to body weight 1 hour after binge alcohol intoxication. (DOCX)

S2 Table Skeletal muscle weights made relative to body weight 24 hours after binge alcohol intoxication. (DOCX)
