# Supplementary material for: Effects of alcohol on skeletal muscle contractile performance in male and female mice
Source: PLoS One. 2021 Aug 12;16(8):e0255946. doi: 10.1371/journal.pone.0255946 (PMC8360553; doi:10.1371/journal.pone.0255946)
Supplement: S1 Table — (DOCX) [file pone.0255946.s002.docx]

**Supplemental table 1:** Skeletal muscle weights made relative to body weight 1 hour after binge alcohol intoxication. Data is presented as mean ± SE.

|  | Female- 1 hour | | Male – 1 hour | |
| --- | --- | --- | --- | --- |
| **Tissue (mg/g)** | **Saline** | **EtOH** | **Saline** | **EtOH** |
| Soleus | 0.40 ± 0.03 | 0.36 ± 0.02 | 0.36 ± 0.03 | 0.34 ± 0.02 |
| Plantaris | 0.64 ± 0.03 | 0.64 ± 0.03 | 0.70 ± 0.02 | 0.71 ± 0.002 |
| Gastrocnemius | 5.10 ± 0.08 | 4.93 ± 0.13 | 4.74 ± 0.16 | 5.04 ± 0.04 |
| Triceps Surae | 6.14 ± 0.07 | 5.94 ± 0.15 | 5.81 ± 0.16 | 6.09 ± 0.05 |
