# Supplementary material for: Effects of alcohol on skeletal muscle contractile performance in male and female mice
Source: PLoS One. 2021 Aug 12;16(8):e0255946. doi: 10.1371/journal.pone.0255946 (PMC8360553; doi:10.1371/journal.pone.0255946)
Supplement: S2 Table — (DOCX) [file pone.0255946.s003.docx]

**Supplemental table 2:** Skeletal muscle weights made relative to body weight 24 hours after binge alcohol intoxication. Data is presented as mean ± SE.

|  | Female- 24 hour | | Male – 24 hour | |
| --- | --- | --- | --- | --- |
| **Tissue (mg/g)** | **Saline** | **EtOH** | **Saline** | **EtOH** |
| Soleus | 0.40 ± 0.01 | 0.41 ± 0.01 | 0.41 ± 0.03 | 0.38 ± 0.02 |
| Plantaris | 0.71 ± 0.02 | 0.68 ± 0.03 | 0.77 ± 0.02 | 0.77 ± 0.03 |
| Gastrocnemius | 5.23 ± 0.11 | 5.25 ± 0.14 | 5.60 ± 0.09 | 5.43 ± 0.17 |
| Triceps Surae | 6.31 ± 0.16 | 6.24 ± 0.14 | 6.78 ± 0.13 | 6.58 ± 0.20 |
